# Assessment of myocardial bridging and the pericoronary fat attenuation index on coronary computed tomography angiography: predicting coronary artery disease risk

**DOI:** 10.1186/s12872-023-03146-6

**Published:** 2023-03-22

**Authors:** Yang Lu, Haifeng Liu, Zuhui Zhu, Siqi Wang, Qi Liu, Jianguo Qiu, Wei Xing

**Affiliations:** Department of Radiology, The Third Affiliated Hospital of Soochow University, Changzhou, Jiangsu 213000 China

**Keywords:** Pericoronary adipose tissue, Fat attenuation index, Myocardial bridging, Coronary artery disease, High-risk plaque

## Abstract

**Background:**

The fat attenuation index (FAI) is a radiological parameter that represents pericoronary adipose tissue (PCAT) inflammation, along with myocardial bridging (MB), which leads to pathological shear stress in the coronary vessels; both are associated with coronary atherosclerosis. In the present study, we assessed the predictive value of FAI values and MB parameters through coronary computed tomography angiography (CCTA) for predicting the risk of coronary atherosclerosis and vulnerable plaque in patients with MB.

**Methods:**

We included 428 patients who underwent CCTA and were diagnosed with MB. FAI values, MB parameters, and high-risk coronary plaque (HRP) characteristics were recorded. The subjects were classified into two groups (A and B) according to the absence or presence of coronary plaque in the segment proximal to the MB. Group B was further divided into Groups B_1_ (HRP-positive) and B_2_ (HRP-negative) according to the HRP characteristic classification method. The differences among the groups were analysed. Multiple logistic regression analysis was performed to determine the independent correlation between FAI values and MB parameters and coronary atherosclerosis and vulnerable plaque risk.

**Results:**

Compared to the subjects in Group A, those in Group B presented greater MB lengths, MB depths and muscle index values, more severe MB systolic stenosis and higher FAI_lesion_ values (all *P <* 0.05). In multivariate logistic analysis, age (OR 1.076, *P <* 0.001), MB systolic stenosis (OR 1.102, *P <* 0.001) and FAI_lesion_ values (OR 1.502, *P <* 0.001) were independent risk factors for the occurrence of coronary atherosclerosis. Compared to subjects in Group B_2_, those in Group B_1_ presented greater MB lengths and higher FAI values (both *P* < 0.05). However, only the FAI_lesion_ value was an independent factor for predicting HRP (OR 1.641, *P <* 0.001).

**Conclusion:**

In patients with MB, MB systolic stenosis was associated with coronary plaque occurrence in the segment proximal to the MB. The FAI value was not only closely related to coronary atherosclerosis occurrence but also associated with plaque vulnerability. FAI values may provide more significant value in the prediction of coronary atherosclerosis than MB parameters in CCTA.

## Introduction

Myocardial bridging (MB) refers to a congenital anomaly in which a coronary artery is partly covered by myocardium. Some studies have demonstrated that the presence of MBs may increase coronary atherosclerosis risk [1] and is also associated with several clinical cardiac events, such as arrhythmia, coronary spasm and myocardial infarction [2–4]. Due to the mechanical compression induced by MB contraction in systole and early diastole, the coronary artery, especially the segment proximal to the MB, may cause disturbed blood flow, which leads to pathological shear stress and contributes to coronary endothelium damage [5]. Eventually, platelet aggregation and lipoprotein deposition contribute to atherosclerosis in the coronary artery proximal to the MB [6]. Currently, coronary computed tomography angiography (CCTA) is considered to be an excellent tool in the noninvasive evaluation of MB parameters due to its effective visualization of MBs [7]. However, the value of radiological MB parameters in predicting coronary atherosclerosis risk is controversial. Some studies have shown that the depth of the MB is an independent risk factor for cardiovascular disease, and subjects with myocardial infarction (MI) were shown to have a significantly greater MB thickness than subjects without MI [8]. However, other studies indicated that the length of the MB, not the depth, was associated with the occurrence mechanism of coronary atherosclerosis [9]. Ishii et al. reported that the MB muscle index (MMI) could provide more significant value in the prediction of coronary plaque burden [10]. Thus, there are still some different viewpoints regarding which MB parameters are better able to predict coronary atherosclerosis risk in MB patients.

In addition, the fat attenuation index (FAI) is also considered to be an imaging marker that can effectively stratify patients with clinical cardiovascular disease. The FAI is a radiological parameter that represents the physiological state of pericoronary adipose tissue (PCAT), and an increase in FAI represents active vessel inflammation [11]. Some studies have shown that a high perivascular FAI value is associated with the prediction of functional myocardial ischaemia and cardiac mortality [12, 13]. Other studies have demonstrated that progression of the noncalcified plaque burden, such as increased necrotic core and fibrofatty volumes, is related to an increase in FAI parameters [14]. Although MB is considered to protect the coronary arteries by the overlying myocardium from the influence of proinflammatory cytokines [15], the coronary artery segment proximal to the MB could still be affected by PCAT inflammation and may result in clinical cardiovascular disease. There is little research on the application of the FAI for coronary atherosclerosis risk stratification in patients with MB. Because PCAT and MB may both have adverse effects on cardiovascular disease, it is necessary to explore their respective abilities in predicting coronary atherosclerosis risk to offer clinicians some early warning information to strengthen interventions for these risk factors and reduce the occurrence of atherosclerosis in patients with MB. In the present study, we aimed to explore and compare the predictive power of FAI and MB parameters in predicting the risk of coronary atherosclerosis in patients with MB.

## Methods

### Study population

**Fig. 1:**
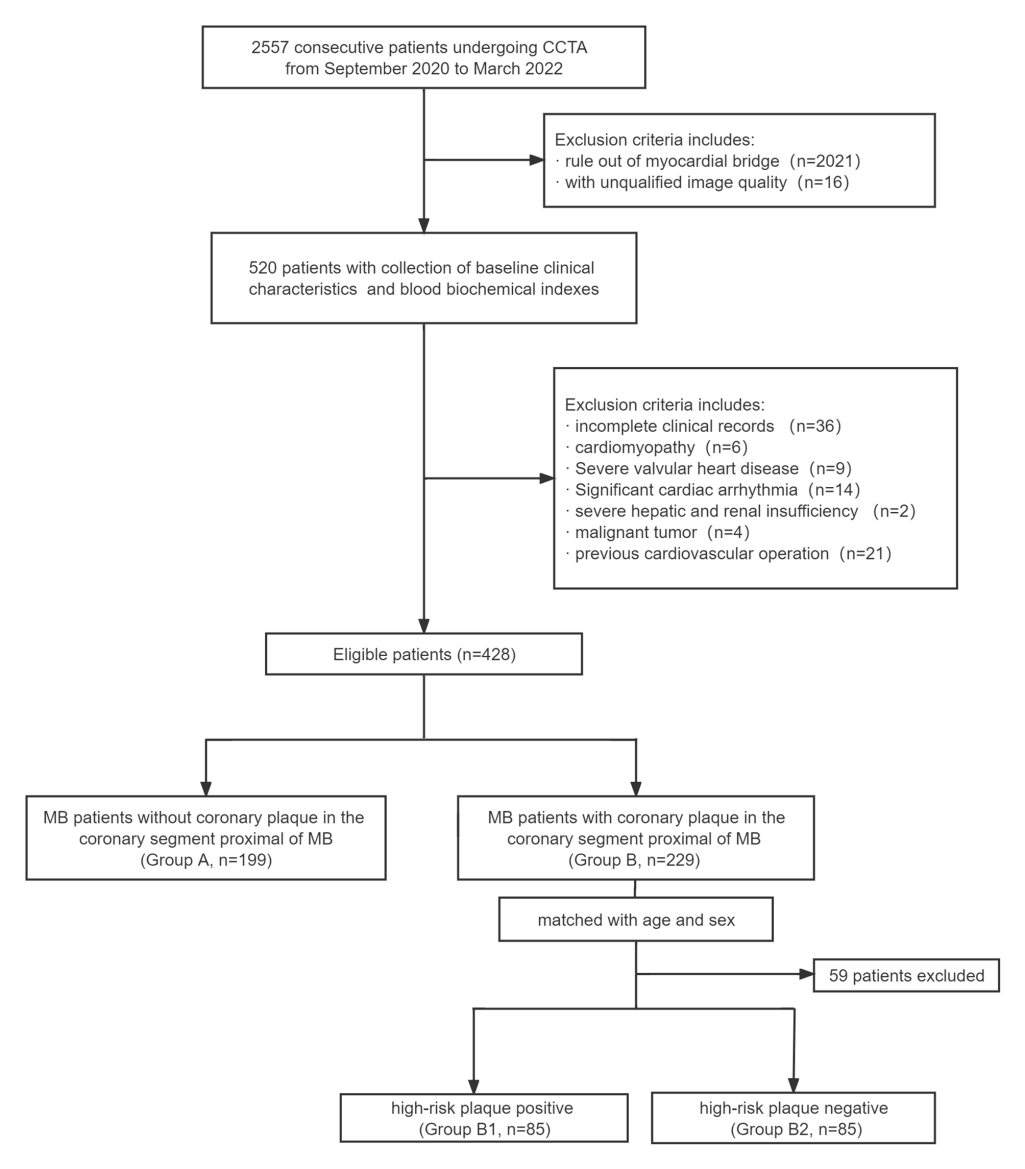
Flowchart of subject enrolment and study design. *CCTA*: coronary computed tomography angiography, *MB*: myocardial bridge

We retrospectively recruited 2557 consecutive patients who underwent CCTA at the Third Affiliated Hospital of Soochow University from September 2020 to March 2022 and confirmed 520 patients with the presence of MB on the left anterior descending (LAD) artery. All patients were referred for evaluation because of chest tightness, precordial discomfort or palpitations. We excluded MB patients with the following conditions: cardiomyopathy (n = 6), severe valvular heart disease (n = 9), significant cardiac arrhythmia (n = 14), severe hepatic and renal insufficiency (n = 2), malignant tumours (n = 4), a previous cardiovascular operation (n = 21), unqualified image quality (n = 16), and incomplete baseline data (n = 36). Ultimately, 428 patients were enrolled in our study (Fig. 1). Then, we collected data on the patients’ baseline clinical characteristics, cardiovascular risk factors and blood biochemical indexes by retrieving their hospital records. The cardiovascular risk factors included a history of hypertension, diabetes mellitus, hyperlipidaemia, smoking and alcohol consumption. The blood biochemical indexes included albumin, glucose, total cholesterol, triglycerides, and high- and low-density lipoprotein. The coronary plaque characteristics, MB parameters, FAI values and PCAT volume of the LAD artery were also collected for each patient after CCTA image reconstruction. Subsequently, we divided the study population into two groups based on the absence or presence of coronary plaque in the segment proximal to the MB (Groups A and B, respectively) to compare the above collected indicators. For further analysis, we also divided the subjects in Group B into two subgroups by the coronary high-risk plaque characteristic classification. Subjects with high-risk coronary plaque (HRP) were included in Group B_1_. Then, from the patients without HRP in Group B, we randomly selected subjects at a 1:1 ratio matched for similar sex and age, who were classified as Group B_2_. Our study was approved by the ethics committee of the Third Affiliated Hospital of Soochow University, Jiangsu, China. Due to the retrospective nature of our study, all examinations were clinically necessary for the patients, so written informed consent was not needed.

### CCTA acquisition and reconstruction

All CCTA examinations were performed using a 256 detector row CT scanner (Revolution CT, GE Healthcare, Waukesha, Wisconsin, USA) with retrospective ECG gating to allow reconstructions at any phase of the R-R interval according to the situation. To ensure image quality, sublingual nitrates were administered to all patients prior to scanning, and those with a heart rate > 75 bpm were also administered beta-blockers. Approximately 55 ml of contrast media (Ultravist 370; Bayer Healthcare, Berlin, Germany) was injected into the antecubital vein with a flow rate of 5.5 ml/s followed by a 30-ml saline flush at the same rate. An automatic bolus tracker was used with a region of interest in the aortic root, and image acquisition was initiated 6 s after an attenuation threshold of 110 HU was attained. The CT images were obtained with the following parameters: a tube voltage of 100 kV or 120 kV, selected by kV assist; a tube current of 350–600 mA, selected by smart-mA technology; a z-coverage of 12–16 cm; a rotation speed of 0.28 s; a field-of-view of 250 mm; a display matrix of 512 × 512; and a detector collimation of 256 × 0.625 mm. The images were automatically reconstructed with 50% adaptive statistical iterative reconstruction-v, and the section interval and thickness were both 0.625 mm. All the data were transferred to a dedicated workstation (ShuKun Technology Co., Ltd., Beijing, China) for further analysis. Reconstructed data were evaluated independently by two radiologists (HFL and YL, both with over 5 years of experience in cardiac CT analysis), and disagreements were resolved by consensus.

### Measurement of MB parameters

The presence of an MB was defined as a segment of the coronary vessel surrounded by cardiac muscle or fibrous tissue. The MB parameters were manually measured through the short-axis and curved multiplanar reformatted images with an electronic calliper on a commercially available workstation (CoronaryDoc; ShuKun Technology). The MB location was defined as the distance between the left coronary ostium and the bridge’s entrance. The MB length was determined by the distance from the entrance to the exit of the tunnelled segment. The depth of the MB was considered to be the perpendicular measurement of the thickness at the deepest point from the tunnelled segment artery to the overlying superficial myocardium. The MMI value was defined as the MB length times the MB depth. MB systolic stenosis was calculated as [(diameter of the coronary artery proximal to the MB - minimal diameter of the MB)/diameter of the coronary artery proximal to the MB] in the end-systole phase [16].

### Coronary plaque analysis

The status of coronary atherosclerotic plaque proximal to the MB was recorded in Group B, including coronary stenosis, high-risk plaque characteristics, the plaque calcification score and plaque length. There are four characteristics of HRP: positive remodelling (PR), napkin-ring sign (NRS), spotty calcification (SPC) and low-attenuation plaque (LAP). The criteria defining each HRP characteristic were based on classifications from previous studies [17]. Patients with at least two of the above features were considered HRP-positive patients. The length of the plaque was manually measured with an electronic calliper in curved multiplanar reformations. The degree of coronary stenosis and the plaque calcification score were automatically calculated on a commercially available workstation (CoronaryDoc; ShuKun Technology).

### Quantification of pericoronary FAI values

**Fig. 2:**
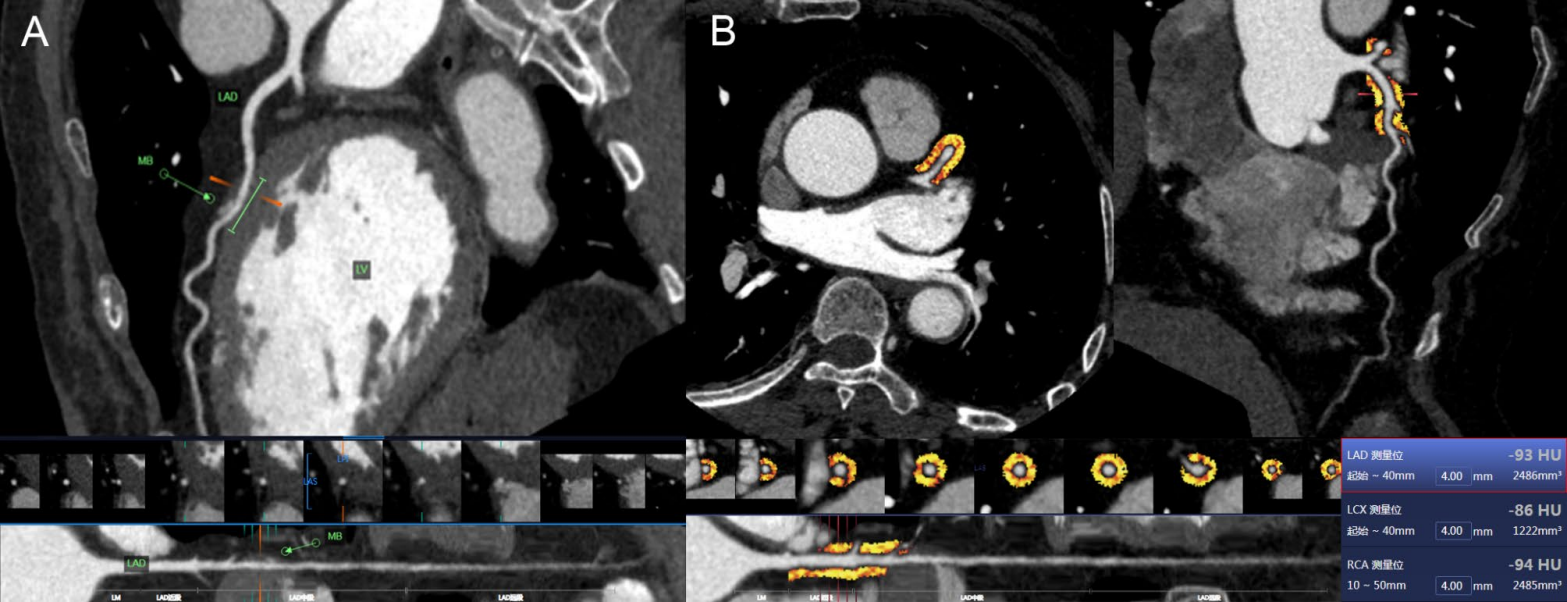
A 62-year-old male underwent CCTA. (**a**) The patient had an MB on the left anterior descending artery, but coronary artery stenosis covered by the MB was not obvious. The patient had no coronary plaque in the segment proximal to the MB. (**b**) The FAI_lesion_ value for the 40-mm segment from the coronary artery proximal to the MB was − 93 HU (the red frame). *CCTA*: coronary computed tomography angiography, *MB*: myocardial bridge, *FAI*: fat attenuation index

**Fig. 3:**
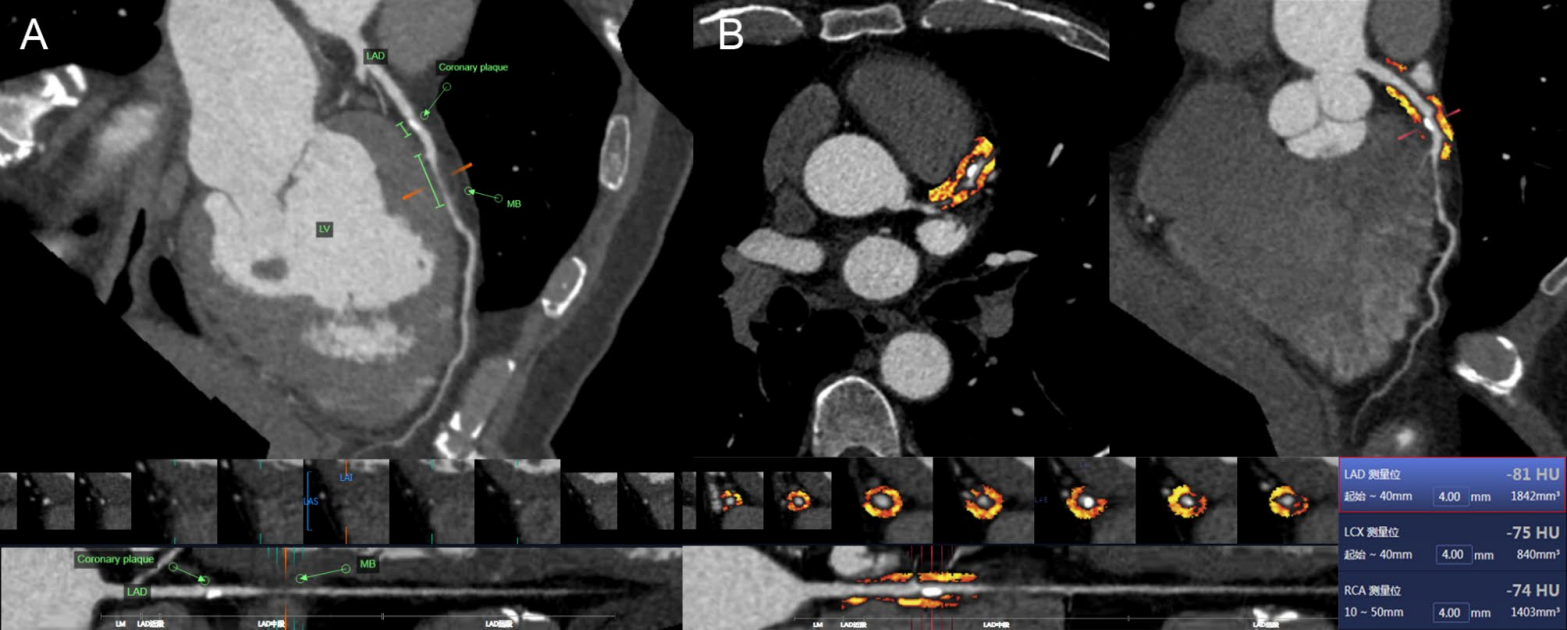
A 68-year-old male underwent CCTA. (**a**) The patient had an MB on the left anterior descending artery, and coronary artery stenosis covered by the MB was obvious. The patient had coronary plaque in the segment proximal to the MB. (**b**) The FAI_lesion_ value for the 40-mm segment around the coronary plaque proximal to the MB was − 81 HU (the red frame). *CCTA*: coronary computed tomography angiography, *MB*: myocardial bridge, *FAI*: fat attenuation index

**Fig. 4:**
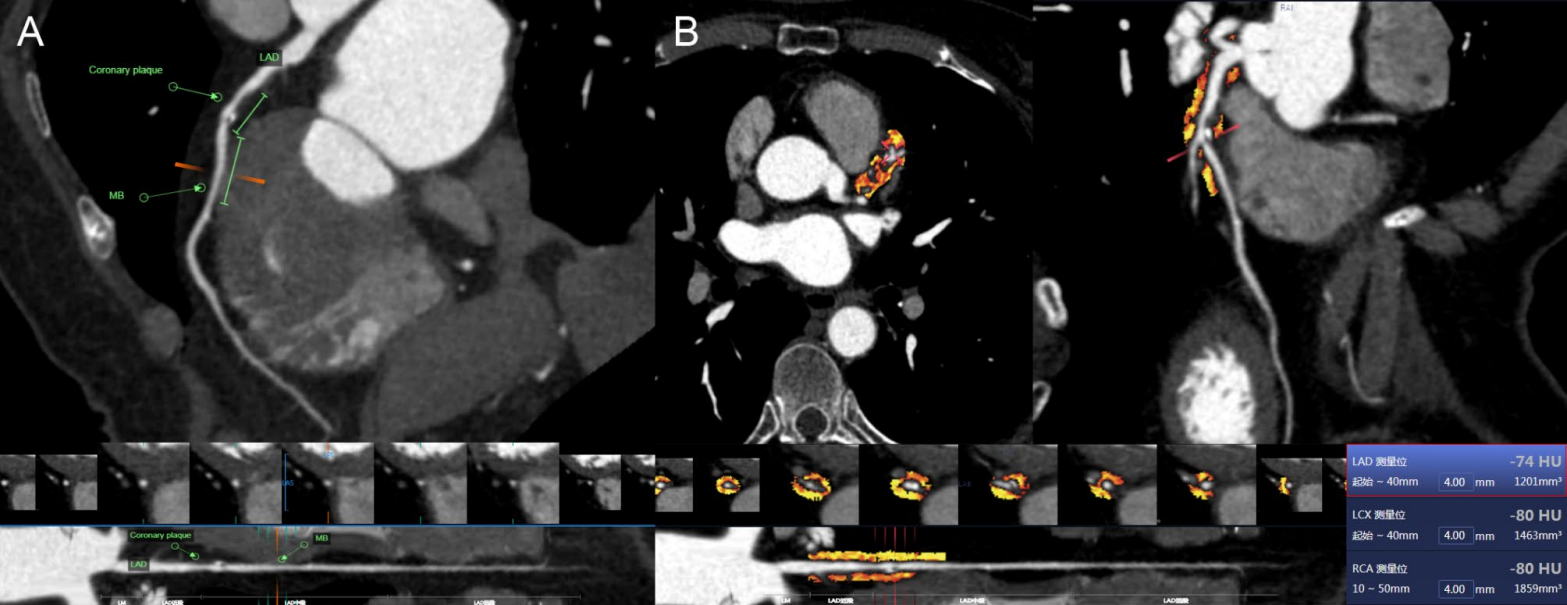
A 65-year-old male underwent CCTA. (**a**) The patient had an MB on the left anterior descending artery, and coronary artery stenosis covered by the MB was not obvious. The patient had high-risk plaque in the segment proximal to the MB. (**b**) The FAI_lesion_ value for the 40-mm segment around the coronary plaque proximal to the MB was − 74 HU (the red frame). *CCTA*: coronary computed tomography angiography, *MB*: myocardial bridge, *FAI*: fat attenuation index

The pericoronary FAI attenuation and PCAT volume parameters in all patients were assessed using semiautomated software (FAI Application of CoronaryDoc; ShuKun Technology). PCAT was demonstrated as all voxels (attenuation thresholds of -190 to -30 HU) located within a distance from the outer vessel wall equal to the diameter of the coronary artery [18]. The FAI value was automatically calculated as the mean CT attenuation value of PCAT, and the PCAT volume was defined as the total volume of adipose tissue containing voxels [19]. For the FAI value and PCAT volume, we specifically measured 40-mm segments from the coronary artery proximal to the MB in Group A and 40-mm segments around the coronary plaque proximal to the MB in Group B. The above FAI values were defined as the FAI_lesion_ values. We also measured a 40-mm segment around the MB in Group B, which was defined as the FAI_MB_ values. Figures 2, 3, 4 demonstrates how the FAI value and PCAT volume were measured.

### Statistical analysis

The data in our study were analysed using SPSS 23.0 (SPSS Inc., IBM Corp., IL, USA). Continuous data are described as the mean ± standard deviation or median. The Kolmogorov–Smirnov test was used to test the normality of the data. For the continuous variables following a normal distribution, the independent samples *t* test was used to assess the intergroup comparisons. Otherwise, the Mann–Whitney U test was applied. Categorical variables are presented as numbers and percentages, and a chi-square test was used to compare the differences between the groups. The paired-samples *t* test was used to identify differences in the FAI_lesion_ and FAI_MB_ values. Univariate and multivariate logistic regression analyses were performed to evaluate the independent association of FAI values and MB parameters with the occurrence of coronary atherosclerosis and vulnerable plaque. A *P* value < 0.05 was considered statistically significant.

## Results

Our study subjects consisted of 428 patients. There were 199 patients in Group A and 229 patients in Group B. For HRP characteristics, the rates of PR, LAP, SPC and NRS were 34.06%, 9.17%, 25.33% and 21.83%, respectively. After recording the HRP characteristics of the patients in Group B, there were approximately 85 patients who were HRP-positive and included in Group B_1_. The mean dose–length product (DLP) of the CCTA was 151.21 ± 49.02 mGy cm, and the mean effective dose (ED) was 2.11 ± 0.68 mSv (DLP multiplied by 0.014 mSv/[mGy cm]). The detailed baseline patient characteristics, biochemical indexes and imaging markers of the subjects and different subgroups are summarized in Table 1.

**Table 1:** Baseline characteristics, laboratory and CCTA findings of all subjects and subgroups

	All patients (n = 428)	Group A(n = 199)	Group B (n = 229)	*P* Value	Group B1 (n = 85)	Group B1 (n = 85)	*P* Value
**Baseline characteristics**							
Age (years)	62.38 ± 10.62	59.21 ± 10.27	65.14 ± 10.16	**< 0.001**	63.05 ± 10.51	65.96 ± 9.65	0.061
Male (%)	254 (59.2)	98 (49.2)	156 (68.1)	**< 0.001**	59 (69.4)	57 (67.1%)	0.742
Height (cm)	165.76 ± 7.82	165.75 ± 7.82	165.76 ± 7.83	0.989	166.76 ± 7.21	165.26 ± 8.22	0.206
Weight (kg)	69.52 ± 12.21	69.36 ± 12.39	69.66 ± 12.05	0.797	70.86 ± 12.84	69.19 ± 11.06	0.365
BMI (kg/m^2^)	25.17 ± 3.23	25.09 ± 3.24	25.23 ± 3.23	0.667	25.31 ± 3.35	25.29 ± 3.18	0.974
Systolic pressure (mmHg)	139.11 ± 18.74	135.43 ± 18.13	142.31 ± 18.71	**< 0.001**	142.94 ± 18.50	142.71 ± 19.88	0.936
Diastolic pressure (mmHg)	81.80 ± 11.74	82.29 ± 12.05	81.37 ± 11.48	0.419	82.02 ± 12.11	81.59 ± 11.84	0.813
Hypertension (%)	289 (67.52)	111 (55.8)	178 (77.7)	**< 0.001**	62 (72.9)	70 (82.4)	0.141
Diabetes mellitus (%)	189 (44.16)	70 (35.2)	120 (52.4)	**< 0.001**	47 (55.3)	45 (52.9)	0.758
Hyperlipidemia (%)	227 (53.04)	102 (51.3)	108 (47.2)	0.398	41 (48.2)	40 47.1)	0.878
Smoking (%)	103 (24.07)	34 (17.1)	70 (30.6)	0.546	25 (29.41)	23 (27.1)	0.733
Alcohol (%)	62 (14.49)	27 (13.6)	36 (15.7)	0.507	9 (10.6)	16 (18.8)	0.130
**Biochemical indexes**							
Albumin (g/L)	40.28 ± 4.29	41.28 ± 3.88	39.42 ± 4.45	**< 0.001**	38.74 ± 4.07	40.22 ± 4.98	**0.035**
Glucose (mmol/L)	6.36 ± 2.76	5.87 ± 1.99	6.78 ± 3.22	**0.001**	6.61 ± 2.99	7.10 ± 3.60	0.335
Total cholesterol (mmol/L)	4.65 ± 1.05	4.89 ± 0.99	4.45 ± 1.07	**< 0.001**	4.48 ± 1.11	4.39 ± 1.13	0.588
Triglyceride (mmol/L)	1.92 ± 1.43	2.11 ± 1.64	1.76 ± 1.21	**0.012**	1.74 ± 1.42	1.79 ± 1.16	0.801
High-density lipoprotein (mmol/L)	1.11 ± 0.27	1.14 ± 0.26	1.08 ± 0.27	**0.014**	1.05 ± 0.25	1.09 ± 0.29	0.411
Low-density lipoprotein (mmol/L)	2.68 ± 0.82	2.82 ± 0.80	2.57 ± 0.83	**0.002**	2.64 ± 0.84	2.50 ± 0.86	0.294
**Coronary plaque measures**							
Coronary artery stenosis (%)	—	—	34.40 ± 19.84	—	33.08 ± 19.29	37.95 ± 21.32	0.120
Coronary plaque length (cm)	—	—	1.15 ± 0.87	—	1.24 ± 0.68	1.22 ± 1.09	0.889
Coronary plaque calcification score	—	—	131.33 ± 185.28	—	70.86 ± 121.55	206.68 ± 243.18	**< 0.001**
Positive remodeling	—	—	78(34.06)	—	64(75.29)	9(10.59)	**< 0.001**
Low-attenuation plaque	—	—	21(9.17)	—	20(23.53)	4(4.71)	**< 0.001**
Spotty calcification	—	—	58(25.33)	—	46(54.12)	6(7.06)	**< 0.001**
Napkin-ring sign	—	—	50(21.83)	—	49(57.65)	1(1.18)	**< 0.001**
**Myocardial bridge measures**							
MB location (cm)	4.37 ± 1.38	4.36 ± 1.30	4.37 ± 1.46	0.972	4.20 ± 1.32	4.52 ± 1.61	0.152
Length of MB (cm)	2.19 ± 1.16	2.01 ± 1.07	2.35 ± 1.22	**0.002**	2.53 ± 1.15	2.14 ± 1.25	**0.035**
Depth of MB (cm)	0.22 ± 0.31	0.16 ± 0.10	0.28 ± 0.41	**< 0.001**	0.32 ± 0.49	0.23 ± 0.13	0.104
MB muscle index	0.52 ± 0.56	0.37 ± 0.31	0.65 ± 0.68	**< 0.001**	0.73 ± 0.71	0.58 ± 0.65	0.143
MB systolic stenosis (%)	35.17 ± 17.89	23.21 ± 12.87	45.56 ± 14.92	**< 0.001**	43.75 ± 16.31	44.24 ± 19.37	0.859
**PCAT measures**							
FAI_lesion_ (HU)	-84.74 ± 8.05	-88.06 ± 7.22	-79.77 ± 6.47	**< 0.001**	-74.06 ± 4.45	-83.07 ± 4.31	**< 0.001**
PCAT volume (cm^3^)	1816.74 ± 458.85	1882.49 ± 425.59	1663.32 ± 433.44	**< 0.001**	1477.44 ± 390.93	1757.93 ± 419.26	**< 0.001**
FAI_MB_ (HU)	—	—	-87.83 ± 7.89	—	-84.34 ± 7.78	-89.72 ± 7.04	**< 0.001**

*CCTA* coronary computed tomography angiography, *BMI* body mass index, *MB* myocardial bridge, *PCAT*PCAT peri-coronary adipose tissue, *FAI* fat attenuation index,

*Group A*: subjects without coronary plaque in the segment proximal to the MB. *Group B*: subjects with coronary plaque in the segment proximal to the MB. *Group B1*: the coronary plaque in the segment proximal to the MB was defined as high-risk coronary plaque. *Group B2*: the coronary plaque in the segment proximal to the MB was not defined as high-risk coronary plaque

Significant *P* values (< 0.05) are in bold

## Differences between subjects in groups A and B

Compared to those in Group A, the subjects in Group B were significantly older, had higher systolic pressure and were more likely to be male. The incidences of hypertension and diabetes mellitus were also higher in Group B. In addition, the levels of albumin, glucose, total cholesterol, triglycerides, and high- and low-density lipoprotein were all significantly different between the two groups. Regarding MB parameters, the patients in Group B presented significantly greater MB lengths (2.35 ± 1.22 vs. 2.01 ± 1.07 cm, *P* < 0.05), depths (0.28 ± 0.41 vs. 0.16 ± 0.10 cm, *P* < 0.001) and MMI values (0.65 ± 0.68 vs. 0.37 ± 0.31, *P* < 0.001) and a more severe degree of MB systolic stenosis (45.56 ± 14.92 vs. 23.21 ± 12.87%, *P* < 0.001) than the patients in Group A. In addition, the patients in Group B presented a significantly higher FAI value (-79.77 ± 6.47 vs. -88.06 ± 7.22 HU) but lower PCAT volume (1663.32 ± 433.44 vs. 1882.49 ± 425.59 cm^3^) than the patients in Group A. A detailed comparison of the results is illustrated in Table 1.

## Differences between subjects in groups B_1_ and B_2_

There were no significant differences in the clinical characteristics between Groups B_1_ and B_2_. The patients in Group B_1_ presented a lower level of albumin than the patients in Group B_2_. Regarding MB parameters, only the length of the MB was significantly different between the two groups. The patients in Group B_1_ presented a significantly greater MB length (2.53 ± 1.15 vs. 2.14 ± 1.25 cm, *P* = 0.035) than those in Group B_2_. For the coronary plaque characteristics, Group B_1_ had a significantly lower coronary plaque calcification score than Group B_2_ (70.86 ± 121.55 vs. 206.68 ± 243.18, *P* < 0.001). In addition, Group B_1_ had significantly higher FAI values (-74.06 ± 4.45 vs. -83.07 ± 4.31 HU, *P* < 0.001) and lower PCAT volumes (1477.44 ± 390.93 vs. 1757.93 ± 419.26 cm^3^, *P* < 0.001) than Group B_2_. A detailed comparison of the results is illustrated in Table 1.

## Independent risk factors for the occurrence of coronary atherosclerosis

In univariable logistic regression analysis, the baseline characteristics, MB parameters, FAI values, PCAT volumes, and biochemical indexes with significant differences between Groups A and B from Table 1 were evaluated. The results showed that age, sex, hypertension, diabetes mellitus, hyperlipidaemia, smoking and all biochemical indexes were associated with coronary atherosclerosis (all *P* < 0.05). In addition, the MB length and depth, the MMI value and MB systolic stenosis were found to have obvious effects on the occurrence of coronary plaque (all *P* < 0.05). The FAI_lesion_ values and PCAT volumes also had a significant association with coronary atherosclerosis (all *P* < 0.001). Then, multivariable forwards stepwise logistic regression analysis was performed for all the above significant univariate predictors and demonstrated that only age (OR 1.076, 95% CI 1.038–1.115, *P <* 0.001), MB systolic stenosis (OR 1.102, 95% CI 1.075–1.130, *P <* 0.001) and the FAI_lesion_ values (OR 1.502, 95% CI 1.357–1.661, *P <* 0.001) were independent factors that predicted the occurrence of coronary atherosclerosis. The detailed results are illustrated in Table 2.

**Table 2:** Univariate and multivariable logistic regression analyses of risk factors for occurrence of atherosclerosis

Variable	Univariable			Multivariable		
	Odds ratio	95% CI	*P*-value	Odds ratio	95% CI	*P*-value
Age	1.058	1.037–1.079	**< 0.001**	1.076	1.038–1.115	**< 0.001**
Male	2.202	1.487–3.263	**< 0.001**			
BMI	1.013	0.955–1.075	0.666			
Systolic pressure	0.993	0.977–1.010	0.419			
Hypertension	0.361	0.238–0.549	**< 0.001**			
Diabetes mellitus	2.029	1.374–2.996	**< 0.001**			
Hyperlipidemia	1.702	0.400–0.863	**0.007**			
Smoking	0.468	0.294–0.744	**0.001**			
Alcohol	0.842	0.696–2.038	0.531			
Albumin	0.897	0.855–0.942	**< 0.001**			
Glucose	1.152	1.058–1.255	**0.001**			
Total cholesterol	0.657	0.541–0.798	**< 0.001**			
Triglyceride	0.834	0.720–0.967	**0.016**			
High-density lipoprotein	0.408	0.198–0.838	**0.015**			
Low-density lipoprotein	0.688	0.543–0.873	**0.002**			
MB location	1.002	0.874–1.150	0.972			
Length of MB	1.305	1.097–1.552	**0.003**			
Depth of MB	1.070	1.047–1.095	**< 0.001**			
MB muscle index	4.153	2.339–7.374	**< 0.001**			
MB systolic stenosis	1.112	1.091–1.133	**< 0.001**	1.102	1.075–1.130	**< 0.001**
FAI_lesion_	1.476	1.363–1.597	**< 0.001**	1.502	1.357–1.661	**< 0.001**
PCAT volume	0.998	0.998–0.999	**< 0.001**			

*CI* confdence interval, *MB* myocardial bridge, *PCAT* peri-coronary adipose tissue, *FAI* fat attenuation index,

Significant *P* values (< 0.05) are in bold

## Independent risk factors for plaque vulnerability

In univariable logistic regression analysis, baseline characteristics, MB parameters, FAI values, PCAT volumes, and the biochemical indexes with significant differences between Groups B_1_ and B_2_ from Table 1 were evaluated. The results showed that only the albumin level, length of the MB, FAI_lesion_ value and PCAT volume were associated with high-risk plaque (all *P* < 0.05). Then, multivariable logistic regression analysis was performed for all significant univariate predictors and demonstrated that only the FAI_lesion_ value was an independent factor that predicted HRP (OR 1.641, 95% CI 1.421–1.894, *P <* 0.001). The detailed results are illustrated in Table 3.

**Table 3:** Univariate and multivariable logistic regression analyses of risk factors for plaque vulnerability

Variable	Univariable			Multivariable		
	Odds ratio	95% CI	*P*-value	Odds ratio	95% CI	*P*-value
Age	0.971	0.942–1.002	0.063			
Male	0.897	0.470–1.712	0.742			
BMI	1.002	0.913–1.099	0.974			
Hypertension	1.436	0.678–3.039	0.344			
Diabetes mellitus	0.910	0.497–1.663	0.758			
Hyperlipidemia	1.048	0.522–1.742	0.878			
Smoking	0.797	0.411–1.544	0.501			
Alcohol	1.958	0.813–4.717	0.134			
Albumin	0.929	0.867–0.996	**0.039**			
MB location	0.858	0.695–1.059	0.154			
Length of MB	1.321	1.015–1.720	**0.038**			
Depth of MB	1.010	0.996–1.025	0.176			
MB muscle index	1.421	0.877–2.302	0.153			
MB systolic stenosis	0.998	0.983–1.017	0.858			
FAI_lesion_	1.641	1.421–1.894	**< 0.001**	1.641	1.421–1.894	**< 0.001**
PCAT volume	0.998	0.998–0.999	**< 0.001**			

*CI* confdence interval, *MB* myocardial bridge, *PCAT* peri-coronary adipose tissue, *FAI* fat attenuation index

Significant *P* values (< 0.05) are in bold

## Differences between the FAI_lesion_ and FAI_MB_ values

**Fig. 5:**
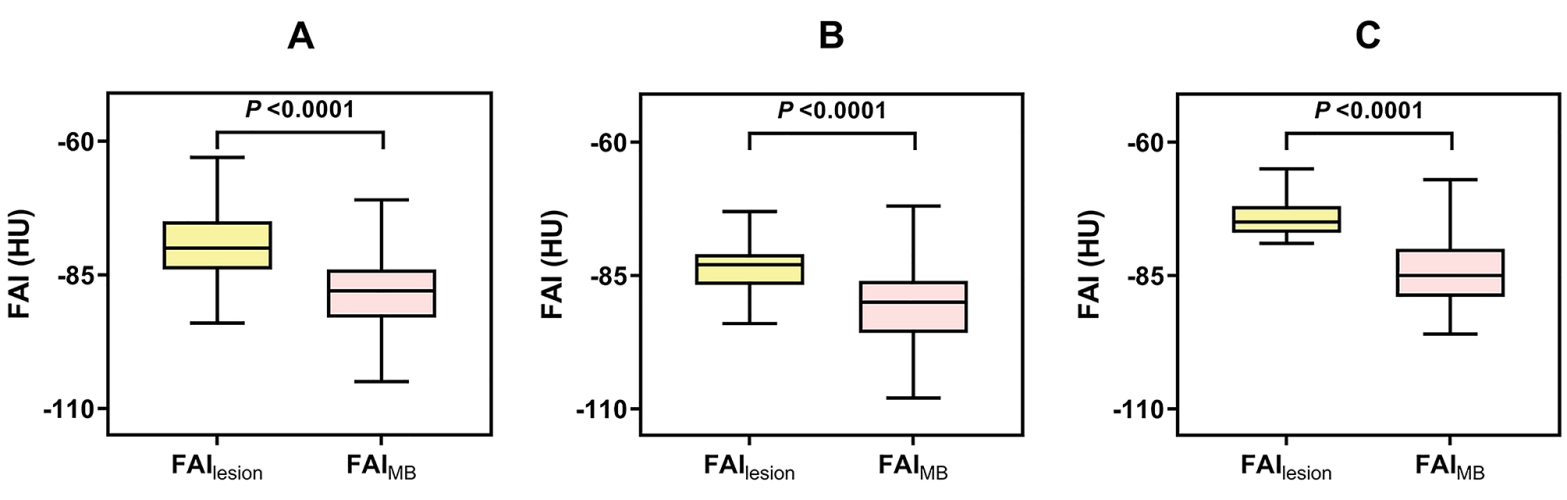
(**a**) Comparison of FAI_lesion_ and FAI_MB_ values in Group B (subjects with coronary plaque in the segment proximal to the MB). (**b**) Comparison of FAI_lesion_ and FAI_MB_ values in Group B_2_ (subjects with HPR negativity in the segment proximal to the MB). (**c**) Comparison of FAI_lesion_ and FAI_MB_ values in Group B_1_ (subjects with HPR positivity in the segment proximal to the MB). *FAI*_*lesion*_: lesion-specific fat attenuation index, *FAI*_*MB*_: myocardial bridge-specific fat attenuation index, *HPR*: high-risk coronary plaque

**Fig. 6:**
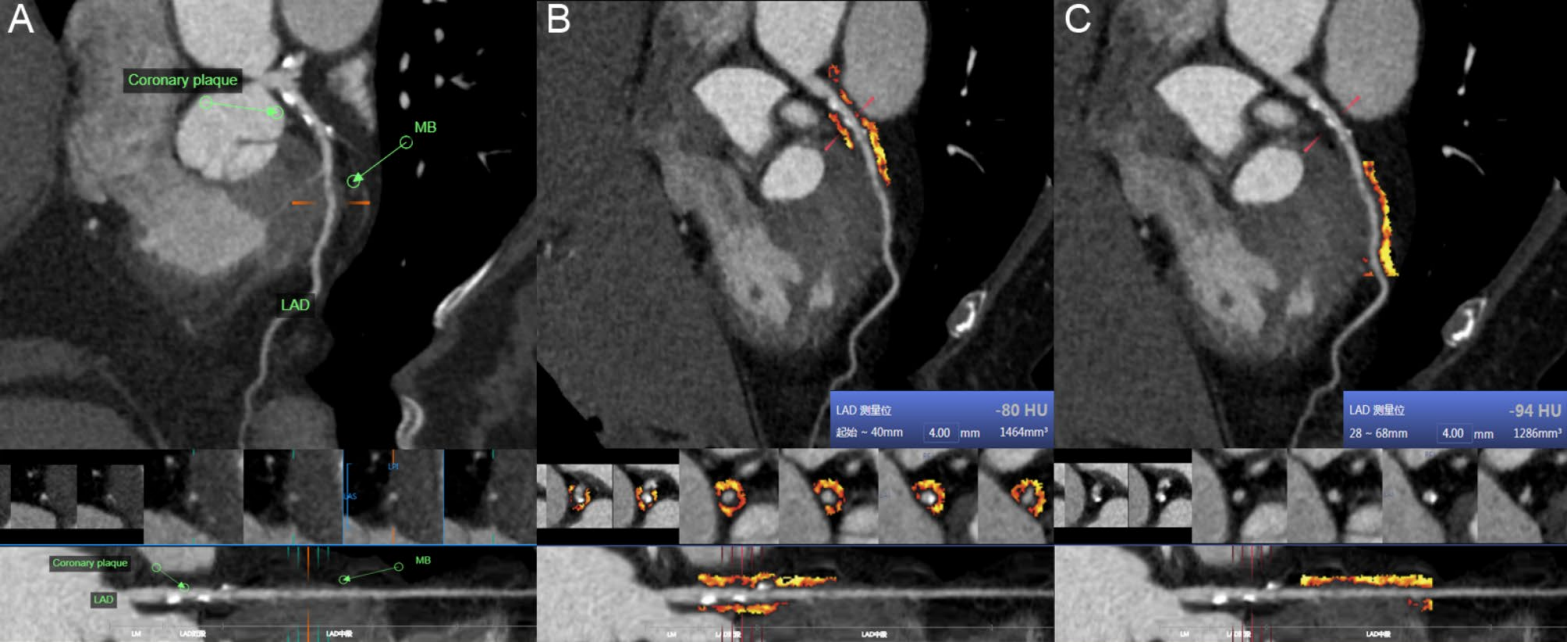
A 59-year-old male underwent CCTA. (**a**) The patient had an MB on the left anterior descending artery and coronary plaque in the segment proximal to the MB. (**b**) The FAI_lesion_ value for the 40-mm segment around the coronary plaque proximal to the MB was − 80 HU. (**c**) The FAI_MB_ value for the 40-mm segment around the MB was − 94 HU. *CCTA*: coronary computed tomography angiography, *MB*: myocardial bridge, *FAI*: fat attenuation index

The difference in Group B is described in Fig. 5a. The FAI_lesion_ value was significantly higher than the FAI_MB_ value (-79.77 ± 6.47 vs. -87.83 ± 7.89 HU, *P* < 0.001). In Group B_1_, the FAI_lesion_ value was significantly higher than the FAI_MB_ value (-74.06 ± 4.45 vs. -84.34 ± 7.78 HU, *P* < 0.001) (Fig. 5c). In Group B_2_, the FAI_lesion_ value was significantly higher than the FAI_MB_ value (-83.07 ± 4.31 HU vs. -89.72 ± 7.04 HU, *P* < 0.001) (Fig. 5b). The specific case for the comparison of the FAI_lesion_ and FAI_MB_ values is shown in Fig. 6.

## Discussion

In this retrospective study, we measured FAI values, MB parameters and coronary plaque characteristics through a single CCTA examination and attempted to examine the factors related to coronary atherosclerosis in patients with MB. Our findings demonstrated that MB systolic stenosis was associated with coronary plaque occurrence in the segment proximal to the MB but was not related to HRP characteristics. The FAI value was closely related to both coronary atherosclerosis occurrence and plaque vulnerability in the coronary segment proximal to the MB. These findings support the potential role of the FAI value and MB systolic stenosis as valid biomarkers of coronary atherosclerosis risk in patients with MB.

Many studies have reported that the presence of MB is closely associated with clinical cardiovascular disease [16]. The most common concept is that MBs in the LAD artery are associated with atherosclerotic lesions proximal to the bridge [20]. The peak systolic intracoronary pressure becomes elevated in the segment proximal to the MB, which reduces systolic antegrade flow, decreases the diastolic/systolic velocity ratio, and causes retrograde flow in the proximal segment [21]. Finally, the LAD intima proximal to the MB is subjected to pathological shear stress. This viewpoint has been confirmed in research with electron microscopy and intravascular ultrasound examinations. The endothelial cell surfaces of the coronary artery in the proximal MB present rough worm-eaten-like defects. Platelets accumulate on the bare basement membrane, causing endothelial cells to detach from the basement membrane, leading to atherosclerotic plaques [22]. Therefore, there are several reasons to believe that the higher systolic compression of MB may have greater effects on the diastolic/systolic velocity ratio and would more easily lead to disturbances in blood flow and endothelial injury. Therefore, in clinical practice, it is necessary to evaluate MB systolic stenosis in patients with MB and consider it a risk factor for coronary atherosclerosis. However, some studies have yielded different findings. Some investigators believe that the location of the MB should be a predictive factor for coronary atherosclerosis. If the MB is closer to the coronary sinus, greater stress on vessels is observed, which is the main factor causing atherosclerosis [23]. In some studies, the total length of the MB was associated with the occurrence mechanism of coronary atherosclerosis. Javadzadegan A et al. reported that an increased length of the MB is associated with decreased wall shear stress and increased residence time [9]. Ishii et al. reported that the MMI (MB length times MB depth) could provide significant value in the prediction of coronary plaque burden [10]. However, these MB parameters showed no independent correlations in our study results. We hypothesize that compared to other MB parameters, MB systolic stenosis is the most significant and direct factor in showing haemodynamic perturbation and the possibility of endothelial injury. Certainly, the contradictory results of previous studies could have been caused by their different measurement and examination methods. Additionally, Ishikawa Y et al. reported that the long-term systolic compression of an MB may lead to coronary plaque rupture at the lesions proximal to the MB segments and result in MI at a younger age [24]. In our study, there were scant data regarding long-term follow-up outcomes to clarify the prognostic role of coronary plaque in MB patients, but it showed that there was no correlation between the presence of an MB and plaque vulnerability. The disturbances in blood flow and endothelial injury in the proximal segment of the MB may have no association with plaque components.

Furthermore, our study also showed that the FAI value was associated with coronary plaque formation, and the risk of plaque formation could even be higher than that of MB systolic stenosis (OR 1.502 vs. OR 1.102). Similar to previous findings, the coronary-specific FAI measurement was regarded as an inflammatory marker to improve cardiovascular risk prediction [25], and increased FAI values represented the pathological state of PCAT inflammation. A wide range of proinflammatory cytokines released from the PCAT diffuse directly into the coronary vessels and eventually contribute to plaque formation [26]. Dang Y et al. reported that FAI value increased along with the severity of coronary artery disease in general and suggested that the FAI value could be an appealing surrogate marker to allow monitoring of PCAT changes [27]. Yan H et al. reported that the combined approach of adding vessel-specific FAI or lesion-specific FAI values could improve the identification of ischaemia compared with CCTA alone [28]. Qin B et al. believed that the peri-stent FAI value can be used as an independent noninvasive biomarker to predict in-stent restenosis risk and severity after stent implantation [29]. Moreover, long-term inflammatory cell infiltration was demonstrated to be not only associated with coronary plaque formation but also a crucial factor in plaque vulnerability [30]. Previous studies suggest that high-risk plaques reveal larger amounts of differentiated subsets of T cells and activated macrophages compared with stable plaques, and they produce larger amounts of proinflammatory cytokines, which would lead to an increase in CT attenuation [31]. Sun JT and colleagues analysed the coronary plaque composition and reported that the increased necrotic core volume and fibrofatty volume are related to an increase in FAI values [14]. Goeller M et al. demonstrated that combined quantitative high-risk plaque features and PCAT CT attenuation may allow for more reliable identification of vulnerable plaques [32]. In the present study, the FAI value was an independent risk factor for plaque vulnerability, even though we eliminated the influence of MB and traditional risk factors. A higher FAI value may not only increase the risk of coronary plaque formation but also increase the risk of high-risk plaque characteristics.

In addition, we also discovered that in patients with coronary plaque in the segment proximal to the MB, regardless of whether the plaque was HRP-positive or negative, the FAI values around the plaque were always higher than the FAI values around the MB, and the presence of plaques was rare in the coronary segment of the MB. This result tends to support the hypothesis that PCAT inflammation may be suppressed in the MB segment, and bridged coronary vessels are protected by the overlying myocardium [15]. The presence of MB could prevent PCAT from coming into contact with the vascular wall, and the MB may protect the vascular wall against proinflammatory cytokines and adipokines secreted by PCAT. In addition, it was interesting that many studies considered the increase in PCAT volume to have a negative impact on cardiovascular function. Balcer B et al. reported that in patients with MI, the PCAT volume was significantly associated with culprit lesions in the underlying coronary artery segments [33]. Ma Y et al. reported that patients with major adverse cardiovascular events (MACEs) had a more significant increase in PCAT thickness at the superior interventricular groove than patients without MACEs [34]. However, only the FAI value, not PCAT volume, had a significant association with coronary plaque occurrence and vulnerability in our study. Under an inflammatory state, PCAT may have an adverse lipotoxic effect and is related to an adverse cardiovascular risk profile [35]. However, under normal physiological conditions, PCAT can also secrete anti-inflammatory cytokines to protect the coronary arteries and supply energy to the myocardium for the storage of fatty acids [36]. Although an increased PCAT volume may have a negative effect on the coronary artery, the PCAT density may better demonstrate the state of inflammation in fat tissue. Wen D et al. also reported that PCAT CT attenuation, but not volume, was related to the haemodynamic significance of coronary artery stenosis [19]. You D et al. reported that risk factors for coronary artery disease and the Gensini score had no association with PCAT volume [37]. The radiodensity of fat tissue may become a more sensitive imaging marker than adipose tissue volume [38]. Furthermore, it is also worth mentioning that among the traditional risk factors and biochemical indexes, only age was an independent influencing factor for coronary plaque occurrence in our study. Several factors may explain this result. First, the subjects in our study were patients with MB, and the highly selected study population may have an influence on the results. The different MB characteristics in different patients may affect plaque occurrence to some degree. Second, our patient sample included many patients with diabetes, hyperlipidaemia and hypertension, and the differences in disease duration and treatments may influence the type of negative effect from the related primary diseases. Third, the effect on coronary atherosclerosis occurs gradually. Regardless of the FAI value or MB systolic stenosis, the longer the endothelial injury is extended in the coronary artery, the easier it is for plaque formation to eventually occur. Hence, we conclude that age is an independent risk factor for coronary atherosclerosis.

## Study limitations

Our study has some limitations that should be pointed out. First, because of its single-centre and retrospective nature and the study population being highly selected, the analysis may be affected by selection bias, limiting the extrapolation of the results to the general population. Furthermore, our study only investigated MBs in the LAD artery because this is the most common location for MBs [39], and our data may not be applicable to MBs in other arteries. In the future, additional data on other coronary branches (the left circumflex artery and right coronary artery) are needed to verify whether the results can be extrapolated to other branches. Third, we used a retrospective scanning protocol because the phases chosen in the prospective studies were limited, but both the cardiac motion artefacts during systolic phases and the administration of beta-blockers to control heart rate may affect the accuracy of the measurements in luminal narrowing in the systolic phase by CCTA. Fourth, each subject had undergone only one imaging exam. Many studies have suggested that intravascular ultrasound [40], coronary magnetic resonance angiography [41] and positron emission tomography/magnetic resonance [42] may also provide information for plaque characterizations. It is necessary for further studies to combine CCTA with these imaging examinations for further exploration. In addition, the use of certain medications may have affected the results. For each subject, different drug regimens may affect their coronary plaque progression. We will try our best to remedy this limitation in future studies. Finally, our study only explored statistical correlations, and multicentre and prospective studies are necessary to provide high-level evidence to make inferences about exact mechanisms and causality.

## Conclusion

In conclusion, our study demonstrated that MB systolic stenosis assessed by CCTA was an independent predictor of coronary atherosclerosis risk. The FAI value had greater predictive power than MB systolic stenosis and was also an independent risk factor for high-risk plaques. In patients with MB, it is plausible that the FAI value and MB stenosis parameters assessed by CCTA may hold potential as noninvasive imaging markers to stratify MB patients and offer clinicians some early warning information to improve the incidence of coronary atherosclerosis.

## Data Availability

The datasets used and/or analysed during the current study are available from the corresponding author upon reasonable request.

## Abbreviations

MB: myocardial bridging
FAI: fat attenuation index
CCTA: coronary computed tomography angiography
MI: myocardial infarction
MMI: myocardial bridge muscle index
PCAT: pericoronary adipose tissue
LAD: left anterior descending
HRP: high-risk coronary plaque
RP: positive remodelling
NRS: napkin-ring sign
SPC: spotty calcification
LAP: low-attenuation plaque
DLP: dose–length product
ED: effective dose
MACEs: major adverse cardiovascular events.
